# Dual effects of human neutrophil peptides in a mouse model of pneumonia and ventilator-induced lung injury

**DOI:** 10.1186/s12931-018-0869-x

**Published:** 2018-09-29

**Authors:** Junbo Zheng, Yongbo Huang, Diana Islam, Xiao-Yan Wen, Sulong Wu, Catherine Streutker, Alice Luo, Manshu Li, Julie Khang, Bing Han, Nanshan Zhong, Yimin Li, Kaijiang Yu, Haibo Zhang

**Affiliations:** 1 Department of Critical Care Medicine, The Second Affiliated Hospital of Harbin Medical University, Harbin, 150000 Heilongjiang China; 2 The State Key Laboratory of Respiratory Disease, The First Affiliated Hospital of Guangzhou Medical University, Guangzhou, 510000 Guangdong China; 3 Keenan Research Center for Biomedical Science of St. Michael’s Hospital, Toronto, ON M5B 1W8 Canada; 4 Department of Critical Care Medicine, The Third Affiliated Hospital of Harbin Medical University, Harbin, 150000 Heilongjiang China; 5 Interdepartmental Division of Critical Care Medicine, Departments of Anesthesia and Physiology, University of Toronto, Toronto, ON M5B 1T8 Canada

**Keywords:** α-Defensins, Neutrophil, P2Y_6_ purinergic receptor, Bacteria clearance, Inflammation, Ventilator-induced lung injury

## Abstract

**Background:**

Pneumonia is a major cause of high morbidity and mortality in critically illness, and frequently requires support with mechanical ventilation. The latter can lead to ventilator-induced lung injury characterized by neutrophil infiltration. The cationic human neutrophil peptides (HNP) stored in neutrophils can kill microorganisms, but excessive amount of HNP released during phagocytosis may contribute to inflammatory responses and worsen lung injury. Based on our previous work, we hypothesized that blocking the cell surface purinergic receptor P2Y_6_ will attenuate the HNP-induced inflammatory responses while maintaining their antimicrobial activity in pneumonia followed by mechanical ventilation.

**Methods:**

Plasma HNP levels were measured in patients with pneumonia who received mechanical ventilation and in healthy volunteers. FVB littermate control and HNP transgenic (HNP^+^) mice were randomized to receive *P. aeruginosa* intranasally. The P2Y_6_ antagonist (MRS2578) or vehicle control was given after *P. aeruginosa* instillation. Additional mice underwent mechanical ventilation at either low pressure (LP) or high pressure (HP) ventilation 48 h after pneumonia, and were observed for 24 h.

**Results:**

Plasma HNP concentration increased in patients with pneumonia as compared to healthy subjects. The bacterial counts in the bronchoalveolar lavage fluid (BALF) were lower in HNP^+^ mice than in FVB mice 72 h after *P. aeruginosa* instillation. However, upon receiving HP ventilation, HNP^+^ mice had higher levels of cytokines and chemokines in BALF than FVB mice. These inflammatory responses were attenuated by the treatment with MRS2578 that did not affect the microbial effects of HNP.

**Conclusions:**

HNP exerted dual effects by exhibiting antimicrobial activity in pneumonia alone condition while enhancing inflammatory responses in pneumonia followed by HP mechanical ventilation. Blocking P2Y_6_ can attenuate the inflammation without affecting the antibacterial property of HNP. The P2Y_6_ receptor may be a novel therapeutic target in attenuation of the leukocyte-mediated excessive host responses in inflammatory lung diseases.

## Background

Bacterial pneumonia is a leading cause of acute respiratory distress syndrome (ARDS) and sepsis contributing to high mortality in the intensive care unit (ICU) [1–3]. During the onset of pneumonia neutrophils are recruited into the infected site and serve as the first line of innate host defense. In patients with pneumonia-associated respiratory dysfunction, mechanical ventilation is an essential supportive approach [4, 5], however, it can lead to ventilator-induced lung injury (VILI). VILI is characterized by neutrophil sequestration that is associated with uncontrolled inflammatory responses [6]. The designate biological consequences of neutrophil infiltration are two folds: 1) Eliminate the pathogens through phagocytosis, and 2) Generate inflammatory responses by production of oxygen radicals, proteolytic enzymes and release of antimicrobial peptides [7].

Pneumonia, ARDS and VILI shares a typical feature of neutrophil infiltration [8–10] acquired for phagocytosis of microbial intruders. Upon completion of phagocytosis the professional phagocytes release granules’ constituents that are strongly anti-microbial but may also cause damage by destructing surrounding tissue. Human neutrophils peptides (HNP) are the most abundant proteins in the azurophilic granules of neutrophils [11], and have been shown to induce inflammatory responses in human lung epithelial and endothelial cells [12–16] and to cause lung injury in mouse models [14, 17] in addition to the microbicidal properties [18]. It is important for one to understand the exact role of HNP in the complex infectious and inflammatory conditions in order to maintain the protective effects while eliminating the unwanted immunological consequences.

HNP, also called α-defensins, are a family of cationic antimicrobial peptides against a broad arrays of pathogens [18]. HNP are synthesized during the promyelocyte stage of neutrophil maturation in bone marrow, and are transported to azurophil granules thus the amount of HNP stored in each granule remains unchanged in mature neutrophils [19], and HNP content is correlated with the number of leukocyte count [20]. Plasma levels of HNP released from neutrophils are under 50 ng/mL in healthy volunteers, but increase up to 4 folds in patients with bacterial infection [21]. HNP concentrations in bronchoalveolar lavage fluid (BALF) can be as high as 50-fold greater in ARDS patients than in healthy volunteers, and are correlated with IL-8 levels in BALF [20]. In patients with cystic fibrosis and mouse model of lung injury, we have previously demonstrated that HNP act on neutrophils and lung epithelium through the purinergic P2Y_6_ receptor [16] resulting in inflammatory responses.

In this study, we employed HNP^+^ transgenic mice to specifically examine the effects of HNP in the models of pneumonia and VILI to reproduce human clinical situations in which large amount of HNP is released [20, 21]. We tested the hypothesis that HNP are protective in pneumonia conditions but are harmful when excessively released in inflammatory conditions when inadequate mechanical ventilation is applied. Blocking P2Y_6_ receptor may attenuate the HNP-induced inflammatory responses without interrupting their antimicrobial activity, serving as a novel therapeutic approach in inflammatory lung diseases.

## Methods

### Plasma collection from pneumonia patients and healthy volunteers

The study was approved by the First Affiliated Hospital of Guangzhou Medical University Institutional Review Board (REC#201311), and written informed consent was obtained from all subjects. Patients with pneumonia admitted to the ICU were consecutively enrolled during October 2012–May 2014. Pneumonia diagnosis was based on clinical features (i.e., fever, dyspnea, increased or purulent secretions, dry and moist rales), chest infiltrates and positive sputum culture [22]. Exclusion criteria were those patients who received chemotherapy within 8 weeks prior to ICU admission. A total of 43 patients (> 18 years) with pneumonia who underwent invasive mechanical ventilation within 24 h of admission were included (Table 1). Age-matched healthy volunteers (*n* = 43) were recruited at physical check-up clinic serving as a control group. Blood samples were collected in EDTA tubes.

**Table 1:** Characteristics of patients with pneumonia and healthy subjects

	Pneumonia (*N* = 43)	Healthy (*N* = 43)
Age (year)	63.8 ± 2.4	63.3 ± 0.7
Female/Male	12/31	19/24
APACHE II	18.1 ± 1.0	
MV mode		
IPPV	44% (*N* = 19)	
SIMV	26% (*N* = 11)	
A/C	14% (*N* = 6)	
Others	16% (*N* = 7)	
VT (mL)	425.9 ± 6.9	
PEEP (cmH_2_O)	6.9 ± 0.5	
PaO_2_/FiO_2_	168.4 ± 10.3	
WBC counts (× 10^9^/L)	14.3 ± 1.2*	7.9 ± 0.3
Neutrophil counts (×10^9^/L)	12.8 ± 1.1*	6.1 ± 0.3
Hemoglobin (g/L)	94.9 ± 2.8*	142.5 ± 1.8
PLT counts (×10^9^/L)	209.6 ± 25.4*	253.9 ± 6.8
Creatinine (μmol/L)	113.8 ± 15.0	
PCT (μg/L)	17.0 ± 6.8	
HSCPR (mg/L)	62.0 ± 13.8	
HNP concentration (ng/mL)	215.9 ± 39.1*	27.6 ± 2.0
Etiology of pneumonia		
Acinetobacter baumannii	35% (*N* = 15)	
Pseudomonas aeruginosa	14% (*N* = 6)	
Stenotrophomonas maltophilia	9% (*N* = 4)	
Staphylococcus haemolyticus	7% (*N* = 3)	
Klebsiella pneumoniae	7% (*N* = 3)	
Staphylococcus aureus	7% (*N* = 3)	
Others	21% (*N* = 9)	
Co-morbidities		
COPD	23% (*N* = 10)	
Hypertension	35% (*N* = 15)	
Diabetes	19% (*N* = 8)	
Renal failure	14% (*N* = 6)	
Asthma	2% (*N* = 1)	
Corticosteroid treatment	37% (*N* = 16)	
ICU stay (days)	28.9 ± 3.5	
ICU mortality (%)	33%	

Note: Data shown are mean ± SEM. * *p* < 0.01 vs. Healthy

*Abbreviations*: *A/C* assist/control, *APACHE* acute physiology and chronic health evaluation, *COPD* chronic obstructive pulmonary disease, *HNP* human neutrophil peptides, *HSCPR* high-sensitivity C-reactive protein, *ICU* intensive care unit, *IPPV* intermittent positive pressure ventilation, *MV* mechanical ventilation, *PCT* procalcitonin, *PEEP* positive end expiratory pressure, *PLT* platelet, *SIMV* synchronized intermittent mandatory ventilation, *VT* tidal volume, *WBC* white blood cell

### HNP endocytosis and IL-8 assays

Human lung epithelial cells (BEAS-2B, ATCC, Manassas, VA) were seeded at 2.5 × 10^5^ cells/slide in chamber slides (Nunc, Naperville, IL), incubated for 30 min at 37 °C with FITC-labeled HNP (10 μg/mL). In additional chamber slides, BEAS-2B cells were treated for 30 min with 10 μM of reactive blue (a purinoceptor (P2Y) antagonist) [23], or suramin (a blocker for receptor-mediated endocytosis), prior to HNP stimulation (100 μg/mL). The cells were then fixed, stained with rhodamine-phalloidin (ThermoFisher Scientific), a high-affinity F-actin probe conjugated to the red-orange fluorescent dye for F-actin, and mounted for visualization.

In separate experiments, BEAS-2B cells or human monocytes (THP-1, ATCC, Manassas, VA) were treated with 10 μM of reactive blue or suramin 30 min before HNP stimulation (100 μg/mL) for 8 h. The supernatants were collected for measurement of IL-8 (MyBioSource, San Diego, CA).

### *P. aeruginosa* culture

*P. aeruginosa* strain (ATCC 27853, Manassas, VA) was incubated overnight at 37 °C on 5% sheep blood agar plates, and colonies were picked and grown overnight in Tryptic Soy Broth (TSB, 5 mL per colony) at 37 °C at 170 rpm (MaxQTM 4500 orbital shaker, Thermo Scientific, Midland, ON, Canada). An overnight cultured bacteria was diluted into TSB, and incubated at a shaker for 2.5 h. The bacteria were then centrifuged at 8000 rpm (J2-MI Centrifuge, Beckman, USA) at 4 °C. After washes with phosphate buffered saline (PBS), the bacterial pellets were resuspended in PBS to a desired density (i.e., an OD value of 0.24 corresponded to 1 × 10^8^ colony formation units (CFU)/mL) determined by spectrophotometry, then the bacterial solution was instilled after 6 times dilutions. The bacterial density used for instillation and collected in the BALF was determined by serial dilutions culture. The culture solution was spread over TSB-agar plates for colony count after incubation overnight.

### Pneumonia and two-hit lung injury models

The animal protocols were approved by the Animal Care Committee of St. Michael’s Hospital. Male FVB mice (Jackson Laboratory, Bar Harbor, ME) served as background control and HNP transgenic (HNP^+^) mice [24] (St. Michael’s Hospital, Toronto, ON) of 10–12 weeks old were anesthesized by intraperitoneal injection of ketamine hydrochloride (50 mg/kg) and xylazine (10 mg/kg), and randomized into either pneumonia (*P. aeruginosa*, P.a.) or vehicle control (PBS) groups. Pneumonia was induced by intranasal instillation of 60 μL *P. aeruginosa* (10^6^ CFU/mouse). This dose of *P. aeruginosa* was chosen based on a pilot study where 0.5 × 10^6^, 1 × 10^6^, 1.5 × 10^6^, 2 × 10^6^ and 5 × 10^6^ CFU/mouse were used and 1 × 10^6^ CFU/mouse resulted in localized bacterial colonization without significant induction of cytokine responses. This would leave a signal gap with respect to cytokine responses and the severity of lung injury between the models of *P. aeruginosa* alone and two-hit injury of *P. aeruginosa* followed by mechanical ventilation.

Following *P. aeruginosa* instillation 48 h later, the mice were either assigned to breathe spontaneously or to receive orotracheal intubation for mechanical ventilation with either a relative protective ventilator strategy of low pressure (LP) ventilation at peak inspiratory pressure (PIP) 10 cmH_2_O, positive end-expiratory pressure (PEEP) 3 cmH_2_O, respiratory rate (RR) 120 breaths/min, or a relative injurious ventilator strategy of high pressure (HP) ventilation at PIP 22 cmH_2_O (to cause overdistension), PEEP 0 cmH_2_O (to cause atelectrauma), RR 70 breaths/min under FiO_2_ of 0.5. Upon completion of 2-h mechanical ventilation, mice were weaned and housed in animal facility for 24 h.

### Administration of P2Y_6_ receptor antagonist

MRS2578 (Sigma, St Louis, MO), a selective P2Y_6_ receptor antagonist, was intraperitoneally injected at a dose of 4 μL/g body weight from a 10 μM stock concentration 15 min after *P. aeruginosa* instillation. Since the half-life of MRS2578 is unknown, we chose to administer MRS2578 at 24 h and 48 h after the first dose, based on previous studies in mice where MRS2578 was effective for 24 h [25–27]. This dose was chosen based on our pilot study testing a dose-dependent response to 10 μM or 20 μM of MRS2578 in HNP^+^ mice after established pneumonia. The triple doses of 10 μM were able to decrease neutrophil infiltration without induction of toxicity. The same volume of 1% DMSO was used to serve as a vehicle control. Based on our pilot studies, we estimated the sample size of 7/group would be sufficient in the present study by using PASS software assuming the expected standard deviation will be 20% of the mean between the groups, with 0.8 of power and α = 0.05. Considering the sample size (i.e., *N* = 5–8 mice/group) being used in other relevant studies [14, 25–28], we finally chose to use 5 mice in PBS control groups, and 7 mice in pneumonia groups in both FVB and HNP^+^ strains.

### Bacterial count, total and differential cell count and albumin in BALF

Upon completion of the study, tracheotomy was performed and a catheter (18G, BD Angiocath, Franklin Lakes, NJ) was inserted into the trachea. A microvessel clamp was used to clip the hilus of the left lung, the right lung was lavaged with three times of injection of 0.5 mL PBS from the catheter. The hilus of the right lung was then ligated and the right lung was excised. The clamp at the left lung was removed and paraformaldehyde was used to inflate the left lung from the catheter. The BALF was serially diluted and cultured for bacterial recovery and counting. The cell pellets were resuspended in PBS for cytospin analysis of cell differentiation. The albumin concentration in the BALF was measured with a mouse albumin ELISA kit (Abcam, Toronto, Ontario, Canada).

### Measurement of HNP and cytokines/chemokines

The HNP levels were determined as previously described [14]. Multiple mouse cytokines and chemokines (TNF-α, IL-1β, IL-6, IL-10, MCP-1 and KC) were measured in BALF by multiplex immunoassay using a Mouse ProcartaPlex Panel (e-Bioscience/Affymetrix, Santa Clara, CA).

### Lung histopathology

The left lung was inflated and fixed with 4% paraformaldehyde and embedded in paraffin. Sections (5 μm) were stained with hematoxylin and eosin (H&E). Lung injury scores were assessed by a pathologist in a blinded fashion using a previously described scoring system based on 8 pathological features: alveolar distention, alveolar collapse, perivascular hemorrhage, perivascular edema, alveolar hemorrhage, alveolar edema, membranes formation and neutrophils infiltration. Each parameter was graded from 0 to 3 [29].

### Statistical analysis

All data are presented as mean ± SEM. The statistical analysis was performed using GraphPad Prism 6. For comparison between pneumonia patients and healthy control groups, the Mann–Whitney U test was used. In vitro study, two-way ANOVA followed by Tukey’s test or Holm-Sidak test was used for statistical comparisons to examine the effect of HNP on IL-8 production, and one-way ANOVA was used for comparisons between reactive blue, suramin and HNP group. Since multiple factors (i.e., mouse strains, bacteria, mechanical ventilation and MRS2578 treatment) were involved in vivo study, significant differences among multiple groups were examined using two-way ANOVA followed by Tukey’s test or Holm-Sidak test. The multiple t-tests were also performed in some comparisons that tended to be significantly different after multiple comparisons for two-way ANOVA. A *p* value < 0.05 is considered statistically significant.

## Results

### Plasma HNP concentrations increased in patients with pneumonia

The demographic and clinical characteristics in patients with pneumonia and healthy subjects are shown in Table 1. There was no significant difference in age between pneumonia patients and healthy donors. Plasma HNP concentration as well as the peripheral white blood cell and neutrophil counts were significantly higher in patients with pneumonia than that in healthy subjects.

### HNP endocytosis by lung epithelial cells

**Fig. 1:**
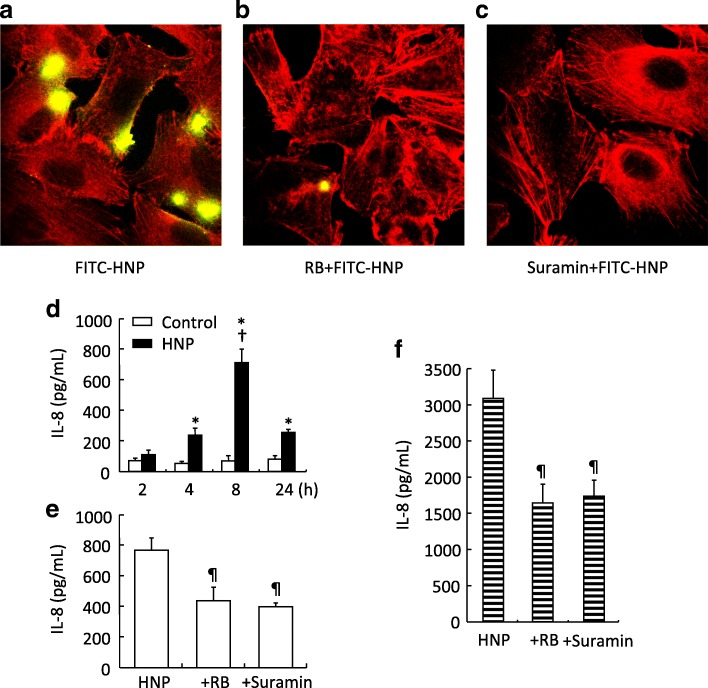
HNP binding to BEAS-2B cells through P2Y receptor family. BEAS-2B cells were incubated with FITC-HNP for 30 min (**a**). HNP are labeled in green and F-actin in red. HNP binding was inhibited by addition of Reactive Blue (RB), or Suramin (**b** and **c**) 30 min prior to FITC-HNP. Effect of HNP on IL-8 production. HNP (100 μg/mL) induce IL-8 production by BEAS-2B cells in a time-dependent manner (**d**). RB (10 μM) or suramin (10 μM) added 30 min prior to HNP administration attenuated IL-8 production in BEAS-2B cells (**e**) and human THP-1 monocytes (**f**) at 8 h. * *p* < 0.05 vs. control group analyzed by Holm-Sidak test; † *p* < 0.05 vs. HNP group at other three time points analyzed by Tukey’s test; ¶ *p* < 0.05 vs. HNP group analyzed by one-way ANOVA, respectively

**Fig. 2:**
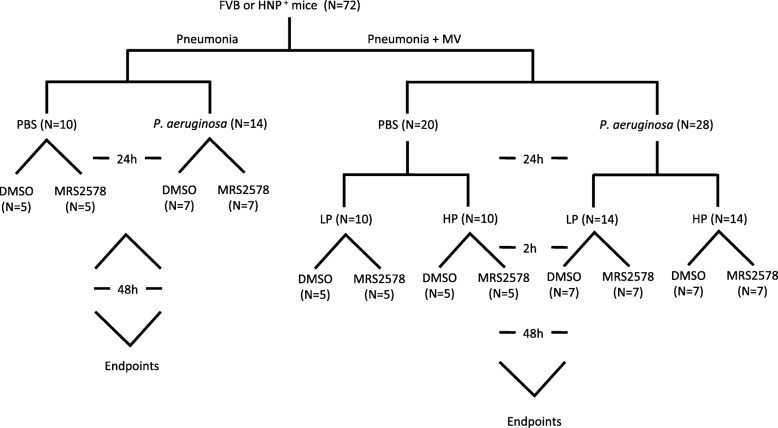
Study design and groups**.** Mice were intranasal instilled with *P. aeruginosa* (1× 10^6^ CFU) or PBS. The mice in two-hit groups were then underwent mechanical ventilation for 2 h at either low pressure (LP) or high pressure (HP). MRS2578 was administrated intraperitoneally 15 min, 24 h and 48 h after pneumonia

When human lung epithelial cells were incubated with FITC-labeled HNP, we observed HNP endocytosis associated with increased IL-8 production (Fig. 1a, d). Both HNP endocytosis and IL-8 production were inhibited by the use of reactive blue and suramin (Fig. 1a-e). The HNP-induced IL-8 production was also attenuated by pretreatment with reactive blue and suramin in the human THP-1 monocytes (Fig. 1f). These results provided us with strong rationale to test the use of P2Y_6_ inhibitor in vivo models (Fig. 2).

### Effects of MRS2578 in model of pneumonia alone

**Fig. 3:**
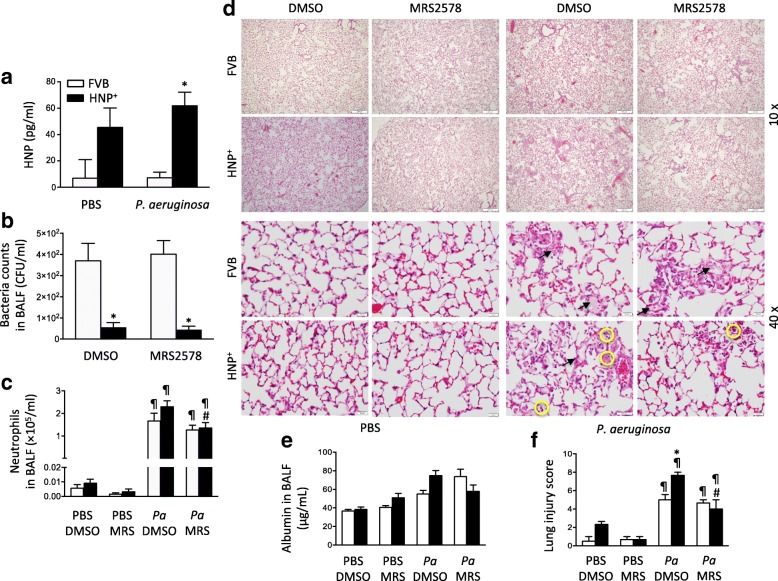
Effects of MRS2578 in pneumonia. Mice were intranasally instilled with *P. aeruginosa* (1× 10^6^ CFU) or PBS. **a** HNP levels in bronchoalveolar lavage fluid (BALF) were measured 72 h later. In additional mice, MRS2578 or vehicle (DMSO) was administered intraperitoneally 15 min, 24 h and 48 h after bacterial instillation. Bacteria counts (**b**) and neutrophils (**c**) in the BALF were determined 72 h after bacterial instillation. **d** Lung histology (H&E) was obtained 72 h after bacterial instillation, Pulmonary edema (black arrow) and neutrophil infiltration (circle) were indicated. **e** Lung permeability was determined by albumin concentration in BALF. **f** Lung injury score. *N* = 7 per group in *P. aeruginosa* groups, *N* = 5 per group in PBS groups. * *p* < 0.05 vs. FVB at identical conditions analyzed by Holm-Sidak test; ¶ *p* < 0.05 vs. PBS at the identical conditions analyzed by Tukey’s test; # *p* < 0.05 vs. DMSO at identical conditions analyzed by Tukey’s test, respectively

HNP expression increased due to high neutrophil count in the HNP^+^ mice despite the fact that there was a similar neutrophil recruitment in both FVB control mice (with no HNP gene) and the HNP^+^ (with HNP transgene) mice in response to *P. aeruginosa* instillation. The plasma HNP levels were higher in the HNP^+^ mice especially in the pneumonia group (Fig. 3a, c). In the pneumonia alone conditions, the BALF bacterial counts were lower in the HNP^+^ mice than in the FVB mice (Fig. 3b), which was associated with neutrophil infiltration in the HNP^+^ mice (Fig. 3c, d). The administration of MRS2578 had no effect in bacterial count (Fig. 3b), but decreased lung injury score in the HNP^+^ mice and not in the FVB mice (Fig. 3d, f).

### Effects of MRS2578 in two-hit model of pneumonia followed by mechanical ventilation

**Fig. 4:**
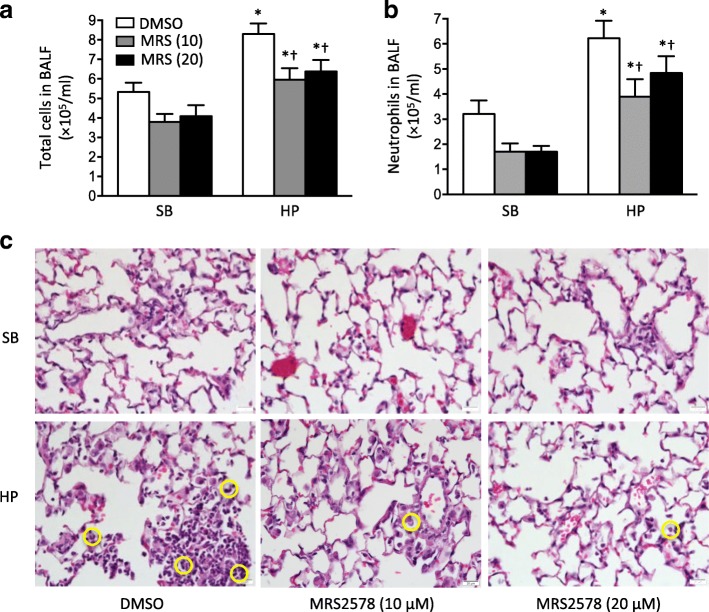
Effects of MRS2578 in HNP^+^ mice with pneumonia followed by mechanical ventilation. HNP^+^ mice were intranasally instilled with *P. aeruginosa* (1× 10^6^ CFU) and then subjected to keep spontaneous breathing (SB) or mechanical ventilation with high pressure (HP) for 2 h. MRS2578 at 10 μM or 20 μM was administrated intraperitoneally 15 min, 24 h and 48 h after pneumonia. Total cell counts (**a**) and neutrophil counts (**b**) in bronchoalveolar lavage fluid (BALF) were determined. **c** Lung histology (H&E) was obtained 72 h after bacterial instillation. Neutrophil infiltration (circle) was indicated. *N* = 7 per group. * *p* < 0.05 vs. spontaneous breathing (SB) at identical conditions analyzed by Holm-Sidak test; † *p* < 0.05 vs. DMSO at identical conditions analyzed by Tukey’s test, respectively

The inflammatory response was attenuated to a similar level following the treatment with MRS2578 at either 10 μM or 20 μM in spontaneous breathing group and in the HP ventilated group after pneumonia in HNP^+^ mice (Fig. 4a-c). Based on this observation, 10 μM MRS2578 was used in the subsequent studies.

**Fig. 5:**
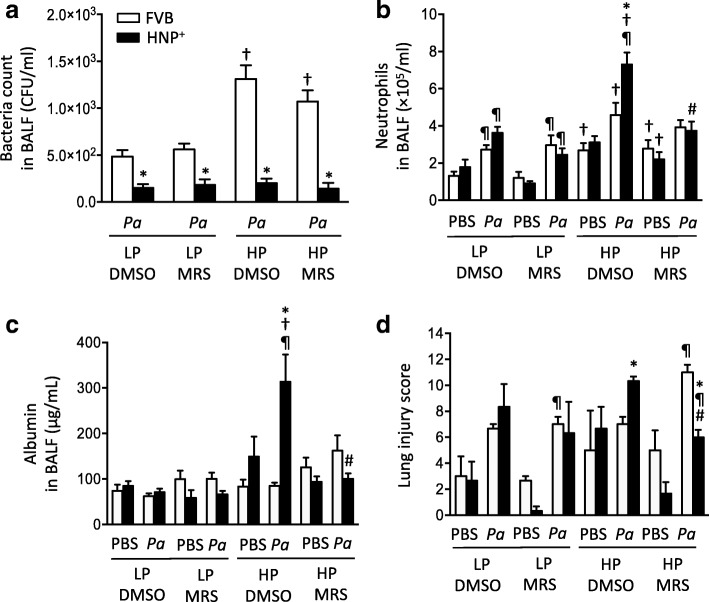
Effects of MRS2578 in pneumonia followed by mechanical ventilation. Mice were intranasally instilled with *P. aeruginosa* (1× 10^6^ CFU) or PBS and received MRS2578 or vehicle (DMSO) 15 min, 24 h and 48 h after pneumonia, and then subjected to mechanical ventilation with either low (LP) or high (HP) pressure for 2 h. Bacteria counts (**a**) and neutrophil counts (**b**) in bronchoalveolar lavage fluid (BALF) were determined. **c** Lung permeability was determined by albumin concentration in BALF. **d** Lung injury score. N = 7 per group in *P. aeruginosa* groups, N = 5 per group in PBS groups. * *p* < 0.05 vs. FVB at identical conditions analyzed by Holm-Sidak test; † *p* < 0.05 vs. LP at identical conditions analyzed by multiple t tests; ¶ *p* < 0.05 vs. PBS at the identical conditions analyzed by multiple t tests; # *p* < 0.05 vs. DMSO at identical conditions analyzed by Tukey’s test, respectively

**Fig. 6:**
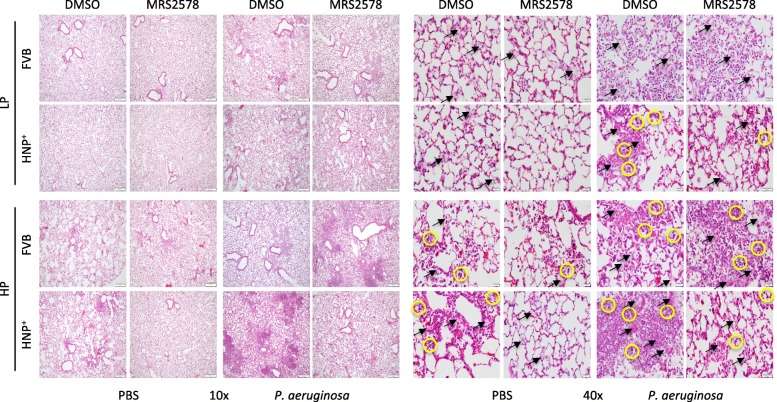
Effects of MRS2578 on histological changes after two-hit lung injury. Mice were intranasally instilled with *P. aeruginosa* (1× 10^6^ CFU) or PBS and received MRS2578 or vehicle (DMSO) 15 min, 24 h and 48 h after pneumonia, and then subjected to mechanical ventilation with either low (LP) or high (HP) pressure for 2 h. Lung histology (H&E) was obtained 72 h h after pneumonia. Pulmonary edema (black arrow) and neutrophil infiltration (circle) were indicated

After *P. aeriginosa* instillation and mechanical ventilation, the bacterial counts remained lower in HNP^+^ mice than in FVB mice, and the use of MRS2578 did not have any effect on bacterial count (Fig. 5a). The application of mechanical ventilation alone did not significantly affect neutrophil infiltration and albumin concentration in BALF (Fig. 5b, c). However, in the two-hit model an increase in inflammatory responses and lung permeability were observed especially in HNP^+^ mice (Fig. 5b, c). The histological analysis showed a trend of increased lung injury score in the HP ventilation groups as compared to the LP ventilation groups but the difference did not reach statistically significance (Figs. 5d and 6). A significant difference was reached in the two-hit injury model in HNP^+^ mice (Figs. 5b-d and 6).

**Fig. 7:**
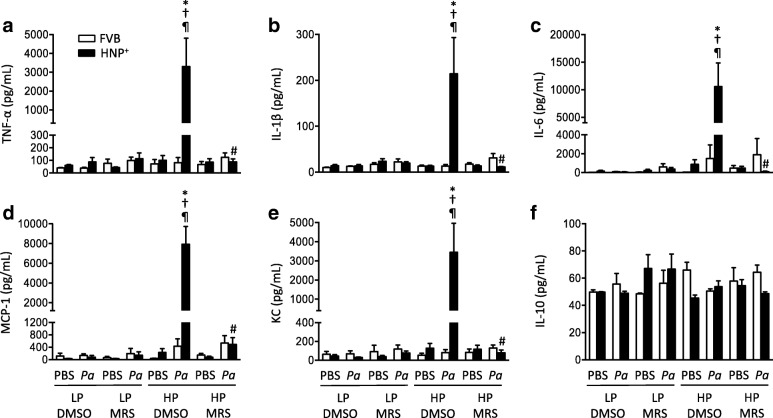
Effects of MRS2578 on production of cytokines/chemokines in two-hit lung injury. Mice were intranasally instilled with *P. aeruginosa* (1× 10^6^ CFU) or PBS and received MRS2578 or vehicle (DMSO) 15 min, 24 h and 48 h after pneumonia, and then subjected to mechanical ventilation with either low (LP) or high (HP) pressure for 2 h. The bronchoalveolar lavage fluid (BALF) was collected 24 h after mechanical ventilation. The concentrations of 6 cytokines/chemokines were simultaneously determined by Luminex multiplex assays. N = 7 per group in *P. aeruginosa* groups, N = 5 per group in PBS groups. * *p* < 0.05 vs. FVB at identical conditions analyzed by Holm-Sidak test; † *p* < 0.05 vs. LP at identical conditions analyzed by Tukey’s test; ¶ *p* < 0.05 vs. PBS at the identical conditions analyzed by Tukey’s test; # *p* < 0.05 vs. DMSO at identical conditions analyzed by Tukey’s test, respectively

The levels of the pro-inflammatory cytokines/chemokines including TNF-α, IL-1β, IL-6, MCP-1 and KC were significantly higher in the HNP^+^ pneumonia mice under HP ventilation than other groups (Fig. 7a-e). The administration of MRS2578 resulted in an attenuation of leukocyte infiltration and lung injury (Figs. 5b-d and 6) as well as cytokine responses (Fig. 7).

## Discussion

Our results demonstrated that HNP concentrations are significantly elevated in pneumonia patients with mechanical ventilation. The experimental studies revealed that HNP play a critical role in the host defense system against *P. aeruginosa* pneumonia, but can also contribute to excessive inflammatory responses in the VILI conditions.

The models of the *P. aeruginosa* induced-pneumonia used in the present study are highly relevant to clinical situations of ARDS [30, 31]. We observed that HNP^+^ mice had much lower bacterial count than FVB mice in pneumonia conditions. This observation is in agreement with the known mechanisms by which HNP exert antimicrobial properties by inducing membrane permeability through the interaction between the positively charged HNP and the negatively charged microorganisms [32, 33], and also inhibiting cell wall synthesis [34].

Although FVB mice do not produce endogenous neutrophil peptides or HNP [35], they do show response to exogenous stimulation with HNP [17, 36, 37]. A previous study has reported greater lung damage by disrupting the capillary-epithelial barrier in HNP^+^ mice than in wild-type mice after the HCl-induced lung injury [28], our study was to examine the effects in a more clinical relevant conditions of pneumonia followed by mechanical ventilation. The protective effects of HNP seen in the pneumonia alone condition have turned into deleterious effects during an overwhelming inflammatory situation under two-hit injury. There are several potential explanations for this finding. Firstly, it has been shown that HNP can induce neutrophil influx into the lung by producing chemokines in lung epithelial cells [12, 13, 17]. Secondly, we have previously demonstrated that HNP at high concentrations interact with neutrophils resulting in decreased phagocytic capacity of neutrophils [14]. Finally, overproduction of reactive oxygen species by HNP [38] might have likely contributes to excessive inflammatory responses. HNP interact with host cells through P2Y_6_ receptor signaling, the use of P2Y_6_ antisense oligonucleotide resulted in attenuation of the HNP-induced IL-8 production *in* ex vivo human lung epithelial cells [16], which is independent of the cationic property. Our results demonstrate that the administration of MRS2578, a selective inhibitor of P2Y_6_ receptor, was able to attenuate the HNP-mediated inflammatory responses (i.e. reduced levels of cytokines and chemokines) and lung injury during HP mechanical ventilation while maintaining the microbicidal activity. In the absence of HNP in FVB mice, the inflammatory responses persisted following pneumonia while administration of MRS2578 had no effect on the mechanical ventilation-induced inflammatory responses. These results further confirmed the involvement of P2Y_6_ receptor acts as a signal sensor for HNP [16] as well as a therapeutic target.

There are limitations in the present study. Our previous work focused on an in vitro study identifying P2Y_6_ signaling in lung epithelial cells [16]. The present study extended the previous by testing the P2Y signal in human monocytes and the P2Y_6_ specific inhibitor in vivo two-hit model combining pneumonia and mechanical ventilation in order to understand the protective and detrimental effects of HNP in different inflammatory conditions. However, our experimental data does not allow one to suggest a critical concentration of HNP that would distinct protective effects from impairment and thus the exact dosage of P2Y_6_ blockade in clinical conditions. The P2Y_6_ receptor is broadly distributed in all organ tissues in human and mice [39, 40]. Thus we did not examine the expression of P2Y_6_ receptor in our study. The scope of our study was to establish the linkage between HNP and P2Y_6_ receptor by application of MRS2578 in conditions of pneumonia and mechanical ventilation. Future studies maybe required to investigate the expression of P2Y_6_ receptor in different conditions such as pneumonia, mechanical ventilation and sepsis, respectively. It is worth noting that in addition to neutrophils, HNP can also be found in natural killer cells, T cells, and immature dendritic cells [18, 41, 42], although it was unlikely the case in our acute models with dominant innate immunoresponse as opposed to a prolonged adaptive response involving immune cells other than neutrophils. Furthermore, we have previously reported that HNP mediated endothelial-monocyte interaction, foam cell formation and platelet activation [15]. The results obtained from our two-hit model using controlled titration of *P. aeruginosa* followed by mechanical ventilation may not be directly translated into all human situations in which bacterial phenotypes and invasion factors are variable. The present study opens a new avenue to conduct more in-depth clinical studies by including patients with ARDS due to non-infectious and patients with pneumonia who are not under mechanical ventilation.

## Conclusions

In summary, our results suggested that blocking P2Y_6_ receptor signaling can attenuate the neutrophils, specifically, the HNP-mediated inflammatory responses without impairing the antimicrobial properties of HNP in the two-hit model of pneumonia followed by mechanical ventilation. Thus targeting the P2Y_6_ signaling may provide a novel therapeutic strategy to reserve the endogenous antimicrobial effects while attenuating the unwanted inflammatory consequence of HNP in inflammatory lung diseases.

## Abbreviations

ARDS: acute respiratory distress syndrome
BALF: Bronchoalveolar lavage fluid
CFU: Colony formation units
H&E: Hematoxylin and eosin
HNP: Human neutrophil peptides
HNP^+^: HNP transgenic
HP: High pressure
ICU: Intensive care unit
LP: Low pressure
P.a.: *P. aeruginosa*
PBS: Phosphate buffered saline
PEEP: Positive end-expiratory pressure
PIP: Peak inspiratory pressure
RR: Respiratory rate
TSB: Tryptic Soy Broth
VILI: Ventilator-induced lung injury
